# Antimicrobial Resistance in Slums: A Call for Global Action

**DOI:** 10.1002/puh2.70028

**Published:** 2025-01-24

**Authors:** Kenneth Chukwuebuka Egwu, Maryam Abdulkarim, Yusuff Adebayo Adebisi, Maria Fay Nenette Cariaga

**Affiliations:** ^1^ Department of Pharmacy University of Nigeria Teaching Hospital, Ituku‐Ozalla, Enugu State Nigeria; ^2^ Department of Molecular Biology and Biotechnology University of Sheffield Sheffield UK; ^3^ College of Social Sciences University of Glasgow Glasgow UK; ^4^ Office of the Vice President for Planning and Research University of Makati Makati City Philippines

Every year, around 7.7 million deaths are linked to bacterial infections. Out of these, 4.95 million deaths are related to drug‐resistant pathogens, whereas 1.27 million deaths are due to bacterial pathogens that are resistant to available life‐saving antibiotics [1]. This reifies the grave threat posed by antimicrobial resistance (AMR), which disproportionately affects vulnerable and marginalized populations [2], including people living in slums. These resistant microorganisms, often referred to as “superbugs,” continue to proliferate, making infections harder to treat and increasing the risk of disease spread, severe illness, and death.

Population settlement patterns, overcrowding, and polluted environments, particularly in slums, play a significant role in the spread of infectious diseases and AMR [3]. For example, regular outbreaks of infectious diseases, such as leptospirosis, malaria, tuberculosis, and dengue, are not uncommon in rural and urban slums [4]. This is especially concerning as the global population of slum dwellers is rising rapidly and is expected to triple from 1 to 3 billion by 2050 [5].

Inadequate access to water, sanitation, and hygiene (WASH) is associated with increased disease burden and AMR [6]. Individuals in slums face a high risk of contracting diseases such as diarrhea due to poor sanitation [7]. According to a 2023 United Nations World Water Development Report, 2 billion people worldwide lack access to safely managed drinking water, and 3.6 billion lack safely managed sanitation services [8]. This shows that a significant proportion of the global population remains highly vulnerable to health risks associated with poor WASH infrastructure.

The lack of clean water and proper sewage systems in slums creates a hotspot for the spread of AMR. This is evident from a study in Bangwe slum (East Blantyre, Malawi), where researchers found that 52% of the surveyed households were drinking water with a high risk concentration of *Escherichia coli* (>100 *E. coli*/100 *m/z*) [9]. *E. coli* (Enterobacteriales) sits predominantly in the critical group of WHO 2024 priority pathogens and was responsible for the highest number of deaths in 2019 [1, 10]. Another study detected diarrheagenic *E. coli* (DEC) in 58% of children residing in slums, compared to only 17% for the control group (private school children in the same city). Worryingly, 65% of the DEC strains showed resistance to at least one antimicrobial, whereas 45% were resistant to two or more antimicrobials [2]. Similarly, in Kenya, the prevalence of diarrhea was estimated to be 32% in the Kibera slums, compared to 13% in the country's capital (Nairobi) [11]. This disheartening ordeal heightens the pressure to consume more antibiotics in these regions, most of which are obtained without prescription due to stunted access to healthcare and the associated cost where available. Similarly, a study in Nigeria revealed that 62%, 87%, and 75% of drug use in Indigenous, Migrant, and Ibadan slums, respectively, occurred without healthcare provider prescriptions (Figure 1) [12].

**FIGURE 1:**
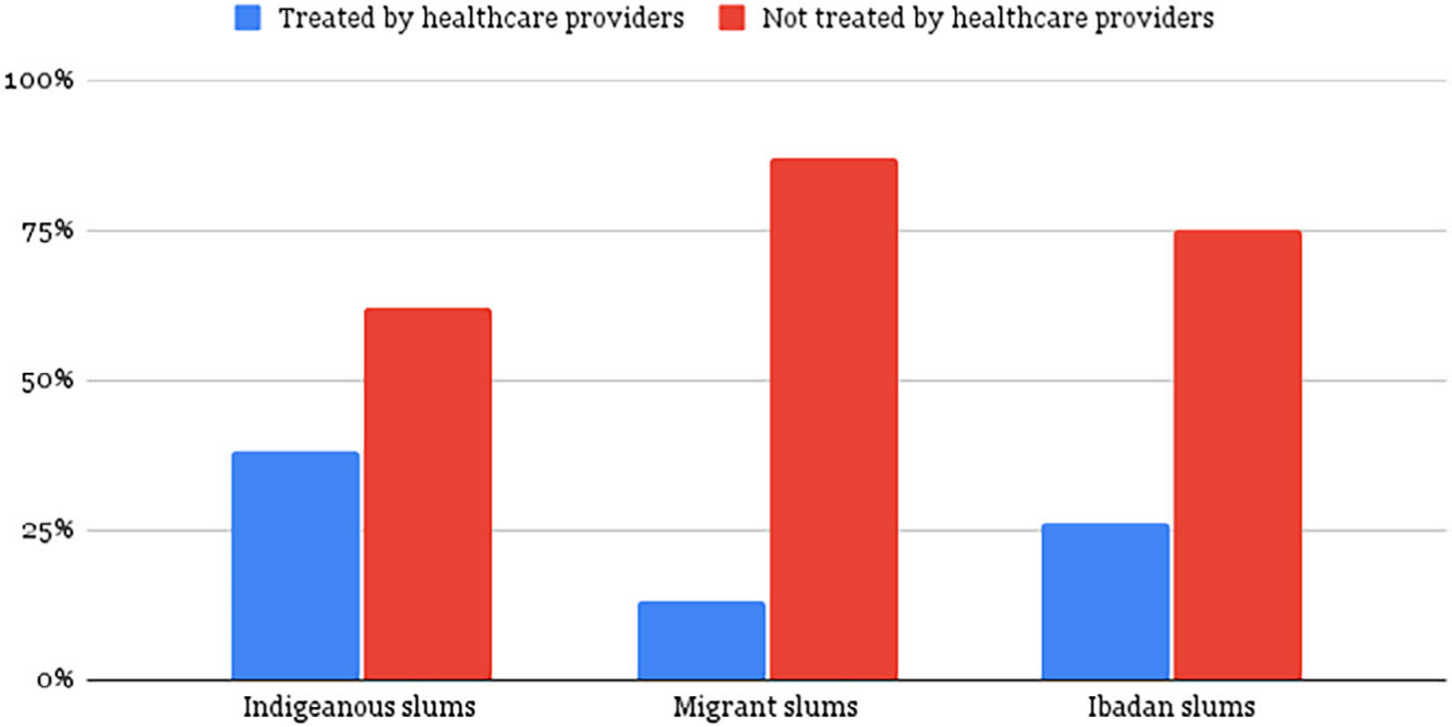
Comparison of Drug Procurement and Treatment Practices Across Indigenous, Migrant, and Ibadan Slums in Nigeria.

It is not surprising that 90% of households in Kibera had used antibiotics in the preceding year, which is a stark contrast to just 17% of typical American households. As a result of this increased antimicrobial use, the Centre for Disease Control and Prevention has reported that typhoid fever cases were 15 times more prevalent among children in Kibera than those living in the rural west area of the capital, with 75% of the identified strain resistant to commonly prescribed antibiotics [13]. In a similar study, resistance to either sulfonamides or co‐trimoxazole was as high as 65% for 79 children aged 5–10 living in slums compared to 16% for similarly aged children attending private schools [14].

The high burden of non‐communicable diseases in slums [15], which predisposes residents to drug‐resistant infections, is compounded by socioeconomic factors such as poverty and low levels of education. These chronic illnesses often weaken the immune system, making individuals more susceptible to infections and complicating treatment options, thereby increasing the likelihood of developing and spreading resistant pathogens. Despite this heightened risk, access to immunization—a critical preventive measure—remains limited for many slum dwellers. For instance, immunization coverage in slums in Niger is just 35% compared to 86% in urban areas, whereas child mortality in Nairobi's slums, where 60% of the population resides, is 2.5 times higher than in other parts of the city [16].

Poverty and lack of education exacerbate the misuse of antibiotics, especially in slums where residents, often excluded from health insurance, may prioritize basic survival over healthcare. As a result, many self‐medicate with antibiotics, risking their health and facing financial ruin when illness worsens. Poverty in slums is wide spread. For example, 70.4% of the population in Devarajeevanahalli slum in Bangalore live below the poverty line [3], a trend mirrored in Purulia slum in India, where 53% of residents are multi‐dimensionally poor [17]. Additionally, the lack of access to medical laboratories and diagnostic tools hinders effective antimicrobial stewardship. Poor infrastructure and inadequate sanitation services in these areas not only increase the transmission of infectious diseases but also drive residents to rely on unregulated, substandard, and counterfeit medications, further contributing to the spread of drug resistance.

Importantly, the potential for drug‐resistant infections to spread from slums to broader communities through water bodies, agricultural produce, and human interaction emphasizes the risk beyond these settlements. This is particularly worrisome as AMR has been projected to cause 10 million annual deaths by 2050 [18]. If this trend continues, the already alarming prediction of AMR costing over $100 trillion globally each year after 2030 could become a reality [18].

Addressing AMR in slums requires a comprehensive approach: transforming slums into habitable communities, providing access to quality healthcare services, expanding health insurance coverage, implementing social empowerment programs to alleviate poverty, ensuring access to education, and regularly sensitizing the population about AMR. Governments, non‐governmental organizations, AMR activists, and healthcare professionals must unite efforts to effectively address AMR in slums. The time to act is now; our collective global action can turn the tide against this silent pandemic, securing a healthier future for all.

## Author Contributions

The article was conceptualized by Kenneth Chukwuebuka Egwu. Kenneth Chukwuebuka Egwu and Maryam Abdulkarim wrote the first draft. Yusuff Adebayo Adebisi and Maria Fay Nenette Cariaga revised the first draft critically for important intellectual content.

## Ethics Statement

The authors have nothing to report.

## Conflicts of Interest

Yusuff Adebayo Adebisi is an editorial board member of Public Health Challenges and co‐author of this article. To minimize bias, he was excluded from all editorial decision‐making related to the acceptance of this article for publication.

## Data Availability

Data sharing is not applicable to this article as no new data were created or analyzed in this study.
